# Pharmacological Preconditioning with Vitamin C Attenuates Intestinal Injury via the Induction of Heme Oxygenase-1 after Hemorrhagic Shock in Rats

**DOI:** 10.1371/journal.pone.0099134

**Published:** 2014-06-13

**Authors:** Bing Zhao, Jian Fei, Ying Chen, Yi-Lin Ying, Li Ma, Xiao-Qin Song, Lu Wang, Er-Zhen Chen, En-Qiang Mao

**Affiliations:** 1 Department of Emergency Intensive Care Unit, Ruijin Hospital, Shanghai Jiaotong University School of Medicine, Shanghai, China; 2 Department of Surgery, Ruijin Hospital, Shanghai Jiaotong University School of Medicine, Shanghai, China; 3 Department of Emergency Intensive Care Unit, the Third People's Hospital, Shanghai Jiaotong University School of Medicine, Shanghai, China; Temple University, United States of America

## Abstract

Pre-induction of heme oxygenase (HO)-1, which is regarded as an effective method of “organ preconditioning”, exerts beneficial effects during hemorrhagic shock (HS). However, the available HO-1 inducers exhibit disadvantages such as toxicity or complex technical requirements. Therefore, a safe and convenient HO-1 inducer would be promising and could be exploited in the treatment of foreseeable hemorrhaging, such as prior to major surgery. Here we investigated the effect of vitamin C (VitC), a common antioxidant, on intestinal HO-1 expression and examined whether VitC pretreatment prevented HS related intestinal tissue injuries after HO-1 induction. First, we conducted an in vitro study and found that HO-1 expression in rat intestinal epithelial cells (IEC-6) was induced by non-toxic VitC in a time and concentration dependent manner, and the mechanism was related to the activation of extracellular signal-regulated kinase 1/2 (ERK1/2). Next, we conducted an in vivo study and found that VitC induced intestinal HO-1 protein expression (mainly observed in the intestinal epithelial cells) and HO-1 activity in normal SD rats, and that these HO-1 levels were further enhanced by VitC in a rat model of HS. The HS related intestinal injuries, including histological damage, pro-inflammatory cytokine levels (tumor necrosis factor and interleukin-6), neutrophil infiltration and apoptosis decreased after VitC pretreatment, and this alleviating of organ injuries was abrogated after the inhibition of HO-1 activity by zinc protoporphyrin-IX. It was of note that VitC did little histological damage to the intestine of the sham rats. These data suggested that VitC might be applied as a safe inducer of intestinal HO-1 and that VitC pretreatment attenuated HS related intestinal injuries via the induction of HO-1.

## Introduction

Globally, 50% of trauma mortality occurs in the age of 5 to 44 years globally [1]. Hemorrhagic shock (HS) is reported to be the leading cause of death in trauma patients [2]. HS and subsequent resuscitation is regarded as a systemic ischemic/reperfusion (I/R) injury, during which the intestine acts not only as a site of end-organ, but also a generator of inflammatory mediators via bacterial translocation. Recently, evidence has shown that intestinal epithelial damage is an early event that is related to the development of multi organ dysfunction induced by HS [3]. Pre-exposure of the intestine to temporary sub-lethal stress, known as “organ preconditioning”, has been shown to increase tolerance to I/R injuries [4]. Several methods for “organ preconditioning” have been described, including brief ischemia followed by reperfusion [5], whole-body hyperthermia [6], and chemical induction of a heat shock protein [7].

Heat shock protein 32, known as heme oxygenase-1 (HO-1), represents one of the important self-protective mechanisms. As an acute phase reactant, HO-1 can be highly and rapidly induced by a wide variety of endogenous oxidative stress stimuli [7], [8]. Recently, exogenous induction of HO-1 has been shown to exert an anti-inflammatory effect, which is mediated by the degradation of its substrate, the pro-inflammatory free heme, and by the production of the anti-inflammatory compounds bilirubin and carbon monoxide [9]. However, the available methods of exogenous induction of HO-1 exhibit substantial disadvantages, such as the severe side effects of protoporphyrins and hemin and the complex technical requirements of adenoviral HO-1 gene transfer, which limit their clinical application [10]. Therefore, finding an agent which can induce HO-1 safely and conveniently is a promising step towards realizing “organ preconditioning”, and the prophylactic delivery of such an agent is hypothesized to be beneficial in cases with foreseeable hemorrhaging, such as in major surgeries with a high risk of massive blood loss.

Vitamin C (VitC), as a water soluble antioxidant, effectively eliminates reactive oxidative species (ROS) by reducing ferric ions into ferrous ions, which are Fenton reaction catalysts [11]. Besides eliminating ROS, VitC also contributes to producing ROS and acts as a prooxidant [11]. Recently, VitC has been shown to attenuate organ injury and inhibit inflammatory responses in various I/R conditions such as cardiac arrest [12] and HS [13], but the specific mechanism remains unclear.

Studies on the relationship between HO-1 and VitC are so far limited, and the results are controversial. VitC was found to induce the HO-1 expression in neurons and glial cells [14] and enhance HO-1 expression induced by the heavy metal As^3+^ [15] Murine Hepa 1c1c7 cell lines, but was also shown to attenuate the induction of HO-1 in a sepsis model [16] or by various agents such as moderately oxidized low density lipoprotein [17] and dopamine [18].The latest report by Moretti [19] demonstrated that VitC exerted antidepressant-like effect which was related to the induction of HO-1.

We reported here that VitC led to the profound induction of HO-1 in intestinal epithelial cells, and the specific molecular mechanism of induction is related to the activation of extracellular signal-regulated kinase 1/2 (ERK1/2). Using a rat model of HS, we demonstrated that VitC pretreatment contributed to the improvement of HS related intestinal injuries and the reduction of inflammatory responses, neutrophil infiltration and apoptosis in the intestine, and that these beneficial effects of VitC pretreatment were attenuated after HO-1 activity was inhibited by zinc protoporphyrin-IX (Znpp-IX).

## Materials and Methods

### Animals and cells

This study was carried out in strict accordance with the guidelines for the care and use of laboratory animals established by the Animal Use and Care Committee of the Shanghai Committee on Animal Care. Animal surgical procedures were approved by the Institutional Animal Care and Use Committee (IACUC) at Shanghai Jiao Tong University, Shanghai, China (Permit Number: SCXK [Shanghai] 2008-0016). Outbred male SD rats (male, 6–7 weeks, 250±10 g) were purchased from the Shanghai Laboratory Animal Center of the Chinese Academy of Science and were allowed to acclimate to the facility for 72 hours. The rats were housed in standard cages in temperature-controlled room (25±0.5°C) with a 12∶12-h light/dark cycle. The rats were provided with free access to food and water. To minimizing suffering, the surgery and sacrifice were performed under sodium pentobarbital anesthesia (50 mg/kg of body weight).

The rat intestinal epithelial cells (IEC-6) were obtained from the American Type Culture Collection (CRL-1592, Manassas, VA, USA) and were maintained at 37 °C in a 5% CO_2_ humidified atmosphere in DMEM containing 10% fetal bovine serum and penicillin G (100 U/ml, Gibco, Grand Island, NY, USA), streptomycin (100 µg/ml; Gibco) and bovine insulin (0.1 U/ml; Sigma, St. Louis, MO, USA).

### HS model

The HS model was established as previously described [20] with slight modifications. The rats were anesthetized with sodium pentobarbital (intraperitoneally, 50 mg/kg of body weight). Using sterile techniques, the left and right femoral arteries were dissected. One heparinized polyethylene tube was inserted into the left femoral artery to monitor blood pressure (Powerlab15T, ADInstrument, Australia), and another tube was inserted into the right femoral artery to withdraw blood. After the baseline blood pressure was measured, HS was initiated in each rat by withdrawing blood into a heparinized syringe (10 units/mL) over a period of 15 minutes to obtain a mean arterial pressure of 30 mmHg, which was continuously maintained for 1 hour through further blood withdrawal or by re-infusing the withdrawn blood (average bleeding volume  = 6±0.5 ml). The rats were resuscitated for 15 minutes by returning all of the withdrawn blood, followed by administering Ringer's solution (6±1 ml) as necessary until the blood pressure was restored to the baseline level. The sham rats underwent all of the instrumentation procedures without the blood collection and resuscitation. The body temperature in each rat was continuously monitored and maintained at 37°C. The electrocardiography was measured constantly.

### Experimental design

#### 1. In vitro study

As previous studies [20], [21] have shown the intestinal epithelial cell is the major source of HO-1 after hemorrhagic shock, the IEC-6 cells were selected to investigate the effect of VitC on HO-1 expression and its specific mechanism in vitro. A non-toxic concentration of VitC was determined using a Cell Counting kit (CCK-8; Dojindo, Kumamoto, Japan), as previously described [22]. A time and concentration-dependent expression of HO-1 was observed for 24 hours after VitC treatment. The activation of the mitogen activated protein kinase (MAPK) family including p38MAPK, the extracellular signal-regulated kinase (ERK) 1/2 and the c-Jun N-terminal kinase (JNK) were observed after VitC treatment. To determine which MAPK was responsible for mediating the HO-1 induction by VitC, the corresponding inhibitors including SB203580 (p38MAPK inhibitor, 10 µM; Cell Signaling Technology [CST], Beverly, MA, USA), PD98059 (ERK1/2 inhibitor, 20 µM; CST) and SP600125 (JNK inhibitor, 25 µM; CST) were added at 1 hour before the VitC treatment. The HO-1 expression was observed at 24 hours after VitC treatment.

#### 2. In vivo study

**Figure 1:**
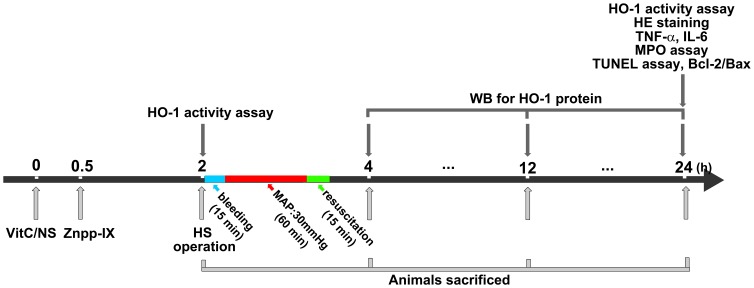
The schematic diagram of the main in vivo protocol. Legend: VitC, Vitamin C; NS, normal saline; Znpp-IX, zinc protoporphyrin-IX; HO-1, heme oxygenase-1; HS, hemorrhagic shock; WB, western blot; HE, hematoxylin and eosin; TNF-α, tumor necrosis factor-α; IL-6, interleukin-6; MPO, myeloperoxidase; TUNEL, TdT-mediated dUTP nick end labeling.

We next investigated the effect of VitC on HO-1 expression in vivo and whether VitC pretreatment prevented intestinal injuries in HS via the induction of HO-1. SD rats were injected with VitC (intraperitoneally, 100 mg/kg of body weight; Sigma) dissolved in normal saline (NS), as described previously [23]. The rats treated with NS were used as the control subjects. Some rats were further treated with ZnPP-IX (intraperitoneally, 3 mg/kg of body weight; Frontier, Logan, UT, USA) at 30 minutes after the VitC treatment as previously described [24]. The jejunum, selected as the representative intestinal segment [25], was harvested at 2, 12 and 24 hours after the VitC or NS administration. In another set of experiments, rats were pretreated with NS (HS group) or VitC (HSV) and subjected to the HS operation 2 hours later. Some rats treated with VitC plus HS operation were additionally given Znpp-IX at 30 minutes after the VitC treatment (HSVZ). The sham rats treated with NS (Sham) or VitC (ShamV) were used as the control subjects. The jejunum segments were harvested at each defined time point (0–24 hours) after the VitC treatment. The HO-1 protein level and activity after HS was observed. The histological changes, the levels of inflammatory cytokines including tumor necrosis factor (TNF)-α and interleukin (IL)-6, the indicators for neutrophil infiltration (number of myeloperoxidase [MPO] positive cells, MPO protein level and activity) and the indicators for apoptosis (number of TdT-mediated dUTP nick end-labeling (TUNEL) positive cells and Bcl-2/Bax ratio) in the intestine were tested. The specific protocol of this part of the study is displayed in **Fig. 1**.

### Western blot

Equal amounts of protein extract (40 µg) were loaded onto a 10% or 12% resolving gel for the electrophoresis. The proteins were trans-blotted onto a Hypond polyvinylidene fluoride membrane (Millipore, Temecula, CA, USA). The membranes were blocked by incubation in 5% nonfat milk for 1 hour at room temperature. The blot was immune-probed overnight at 4°C with the indicated antibodies, including HO-1 (1∶2000; Abcam, Cambridge, MA, USA), β-actin (1∶1000; Santa Cruz Biotechnology, Dallas, TX, USA), MAPK and phosphor-MAPK family antibodies (1∶1000; CST), Bcl-2 (1∶1000; CST), Bax (1∶1000; CST), and MPO (1∶2000; CST). The blots were incubated with an HRP-conjugated secondary antibody for 1 hour at room temperature. The signals were detected by ECL and quantified using Photoshop CS6 software (Adobe, USA).

### Immunocytochemical and immunofluorescence staining

After fixation in 4% paraformaldehyde for 10 minutes and permeation using 0.2% Triton X-100 for 10 minutes, the IEC-6 cells were incubated with the HO-1 primary antibody (1∶200; Abcam) at 4°C overnight. The sections were incubated with CY3-conjugated goat-anti-rabbit immunoglobulin G (1∶200; Jackson, West Grove, PA, USA) at room temperature for 1 hour. All the sections were counterstained with DAPI nucleic acid stain (Invitrogen), and images were taken with an M1 Zeiss microscope under 400X magnification.

### Immunohistochemistry and TdT-mediated dUTP nick end-labeling (TUNEL) staining

The jejunum sections were retrieved in a citrate buffer (0.01 M, pH 6.0) and subjected to heat treatment using a microwave. For the HO-1 and MPO staining, the protocols were subjected to the guidelines of the Histostain-Plus Kits (Invitrogen, Frederick, USA), the slides were blocked with 10% non-immune goat serum for 30 minutes and incubated at 4°C overnight with the indicated antibody, including HO-1 (1∶400; Abcam) and MPO (1∶200; CST). The slides were further incubated with a biotinylated secondary antibody for 1 hour. The TUNEL staining was performed using the In Situ Cell Death Detection Kit according to the manufacturer's instructions (Roche, Mannheim, Germany), and the sections were counterstained with 4′, 6-Diamidino-2- phenylindole (DAPI) nucleic acid stain (Invitrogen). The images were taken with an M1 Zeiss microscope (Jena, Germany). For the neutrophil infiltration evaluation, the MPO positive cells were counted under 400X magnification at 6 “hot spots” per slide; for the apoptosis evaluation, the apoptosis cells and total cells were counted, and the ratio between the counts was expressed as the apoptosis index.

### HO-1 activity assay

As described by Liu et al. [26], HO-1 activity was measured using the spectrophotometric determination of the formation of bilirubin according to the manufacturer's instructions (Genmed Scientifics, Arlington, MA, USA).

### Histological study

The histological study was performed using the hematoxylin and eosin staining method. The histological changes observed in the slides scored by an experienced pathologist blinded for the sample grouping. As described previously [27], the severity of the small intestine injury was scored from 0 to 3 as follows: 0, normal, no damage; 1, mild, focal epithelial edema and necrosis; 2, moderate, diffuse swelling or necrosis of the villi; 3, severe, diffuse necrosis of the villi with evidence of neutrophil infiltration in the submucosa and/or hemorrhage. All of the evaluations were performed on 6 fields per section and 6 sections per organ under 100X magnification.

### Enzyme linked immunosorbent assay

The protein levels of TNF-α and IL-6 were quantified using an enzyme linked immunosorbent assay (ELISA) kit (Mosaic ELISA system, R&D systems, Minneapolis, MN) according to the manufacturer's instructions. The samples were measured in duplicate. The readings from each sample were normalized for the protein concentration.

### Real-time PCR

After the samples were homogenized in liquid nitrogen, TRIzol reagent (Invitrogen) was added to the tissue samples, and 2 µg of total RNA was extracted. The reverse transcription reaction was conducted in a mixture containing random primers, Revert Aid Reverse Transcriptase, RNase inhibitor, and dNTP (Thermo, Lithuania, EU). The PCR reaction mixture was prepared with SYBR Premix Ex Taq (Takara Bio Inc., Shiga, Japan) with specific upstream and downstream primers. The thermal cycling conditions were 10 seconds at 95°C for the initial denaturation step followed by 40 cycles of 95°C for 5 seconds and 60°C for 20 seconds on a real-time PCR system (7500, ABI, Foster, USA). The mRNA levels of TNF-α and IL-6 were expressed relative to the sham rats using the ΔΔCt method. The primer sequences are shown in **Table S1.**

### MPO activity assay

The MPO activity assay was performed as described previously [28], and the MPO activity was expressed in units, where 1 U represents the amount of enzyme degrading in 1 µmol/L H_2_O_2_ per minute. The units of activity were normalized to 1 mg of protein.

### Statistical analysis

All of the data are expressed as the mean ± SEM and compared using the unpaired Student's t-test and a one-way analysis of variance followed by Tukey's test. The differences with a probability value of *p*<0.05 were considered significant. All the statistical calculations were performed using Prism 4 software (GraphPad Software, San Diego, CA, USA).

## Results

### Hemodynamic data of animals during hemorrhage and resuscitation

**Figure 2:**
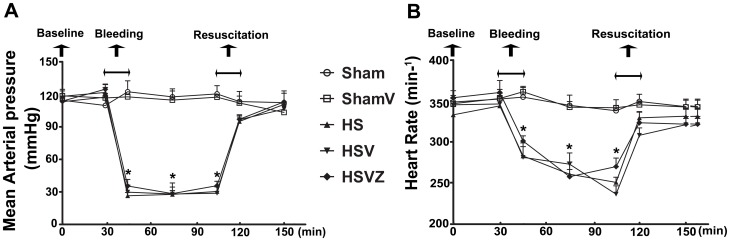
Hemodynamic data of animals during hemorrhage and resuscitation. Sham: the rats treated with normal saline (NS) and the sham operation; HS: the rats treated with NS and the HS operation; HSV: the rats treated with VitC and the sham operation; HSVZ: some rats in the HSV group were additionally treated with Znpp-IX. The mean arterial pressure (MAP, A) and heart rate (HR, B) were shown to decrease during HS and recover to baseline values after resuscitation. Data are mean ± SEM, n = 6/group. *p<0.05 compared to sham.

Baseline values of mean arterial pressure (MAP) and heart rate (HR) were comparable in all groups. The rats in the groups received sham operation exhibited normal and stable hemodynamics including MAP (115.0±7.9 mmHg) and HR (347.4±14.0 beats per minute) throughout the experiment. After bleeding for 15 minutes, the baseline values of MAP (**Fig. 2A**) and HR (**Fig. 2B**) in the groups received HS operation (118.0±7.5 mmHg; 347.2 ±18.5 beats per minute) markedly decreased to a lower level (30.3±5.6 mmHg, 265.9±25.1) which had been maintained for 1 hour. After resuscitation for 15 minutes, the decreased values of MAP and HR gradually returned to the baseline value.

### VitC induced HO-1 expression through ERK1/2 but not p38MAPK or JNK in vitro

**Figure 3:**
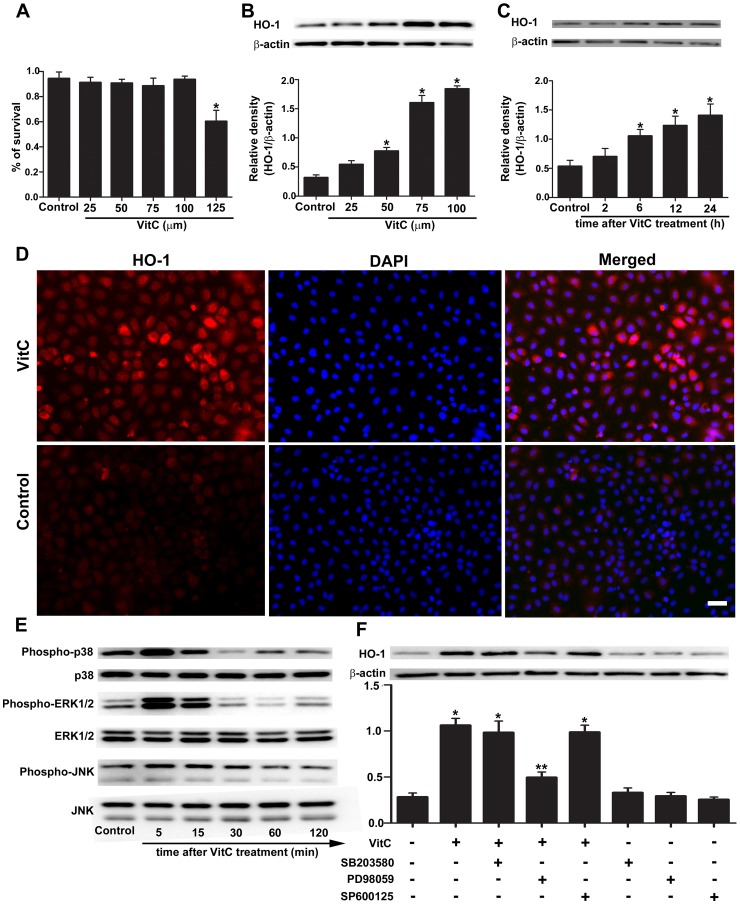
Vitamin C (VitC) induced heme oxygenase (HO-1) expression through extracellular signal-regulated kinase (ERK) 1/2. Control: the IEC-6 cells treated with phosphate buffer solution. A, the cell viability was evaluated by the Cell Counting Kit-8 (CCK-8) at 24 hours after treatment with an increasing dose of VitC (25–125 µM), and the non-toxic concentration of VitC was determined. B, western blot analysis for the HO-1 protein level in the IEC-6 cells at 24 hours after treatment with an increasing dose of non-toxic VitC (25–100 µM). C, western blot analysis for the time-course change of the HO-1 protein level in the IEC-6 cells after the VitC treatment (100 µM). The densitometric analysis in B, C was used to calculate the normalized protein ratio (HO-1 to β-actin). D, immunofluorescent staining of HO-1 (red) in the IEC-6 cells at 24 hours after VitC treatment (100 µM). Magnification: 400X. Scale bar, 50 µm. E, western blot analysis for the time-course change of the phosphorylation extent of the p38 mitogen activated protein kinase (MAPK), ERK1/2 and c-Jun N-terminal kinase (JNK) in the IEC-6 cells after VitC treatment (100 µM). F, western blot analysis for the HO-1 protein level in the IEC-6 cells at 24 hours after VitC treatment (100 µM) in the presence or absence of SB203580 (10 µM), PD98059 (20 µM) and SP600125 (50 µM). Data are mean ± SEM of six independent experiments. *p<0.05 compared to control, **p<0.05 compared to VitC alone.

First, we investigated the effect of VitC on HO-1 expression in IEC-6 cells and the involved mechanism. The CCK-8 assay showed that the maximum nontoxic concentration of VitC was 100 µM (**Fig. 3A**). The HO-1 expression in IEC-6 cells was induced by VitC in a time-dependent (**Fig. 3B**) and concentration-dependent (**Fig. 3C**) manner. The immunofluorescence staining (**Fig. 3D**) showed strong signal of HO-1 were mainly detected in the cytoplasm of IEC-6 at 24 hours after VitC treatment. The p38MAPK, ERK1/2 and JNK were rapidly phosphorylated after VitC treatment (**Fig. 3E**). To identify the MAPK subfamily through which VitC induced HO-1 expression, we used different pharmacological signal inhibitors (SB203580 for p38MAPK, PD98059 for ERK1/2 and SP600125 for JNK) and found that only PD98059 significantly abolished the VitC induced HO-1 expression at 24 hours after VitC treatment (**Fig. 3F**). The signal inhibitors themselves did not affect the HO-1 expression (**Fig. 3F**).

### VitC induced intestinal HO-1 protein level and activity in vivo

**Figure 4:**
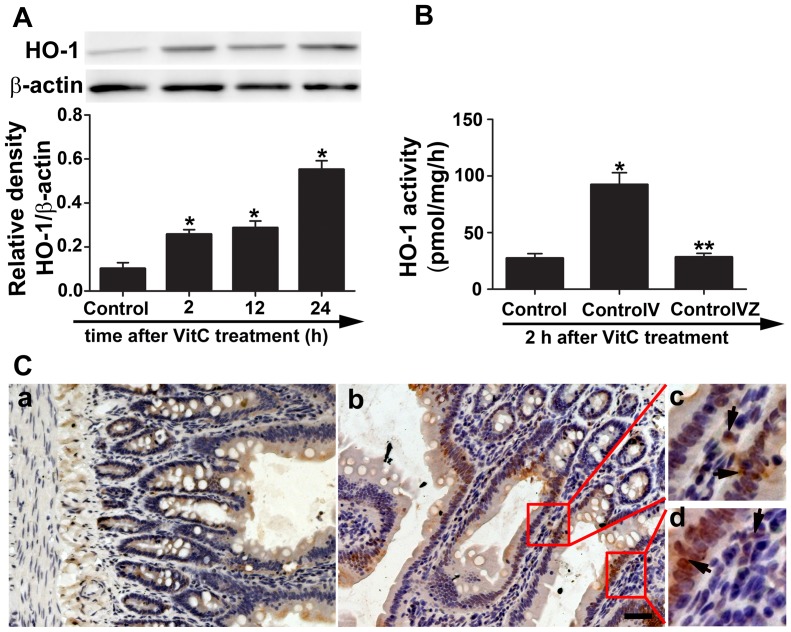
Vitamin C (VitC) induced intestinal heme oxygenase (HO)-1 protein level and activity. Control: the rats treated with normal saline (NS); ControlV: the rats treated with VitC; ControlVZ: the rats treated with VitC and Znpp-IX. A, western blot analysis for the time-course change of the intestinal HO-1 protein level in the normal rats after VitC treatment. B, the intestinal HO-1 activity was measured at 2 hours after VitC treatment. C, the intestinal HO-1 staining in the normal rats at 24 hours after NS (a) or VitC (b, c, d) treatment. The arrows indicate the HO-1 positive cells. Magnification: 200X. Scale bar, 100 µm. Data are mean ± SEM, n = 6/group. *p<0.05 compared to control, **p<0.05 compared to ControlV.

We then tested the effect of VitC on HO-1 expression in normal SD rats. The HO-1 protein level was quickly induced at 2 hours after VitC treatment and gradually increased during the 24 hours after VitC treatment (**Fig. 4A**). The HO-1 activity which revealed that enzymatic function was enhanced at 2 hours after the VitC treatment and the increased HO-1 activity was reduced to the baseline level by Znpp-IX (**Fig. 4B**). The HO-1 protein was predominantly observed in the mucosal intestinal epithelial cells, and to a lesser extent, in the lamina propria cells (**Fig. 4C**).

### VitC enhanced intestinal HO-1 protein level and activity after HS

**Figure 5:**
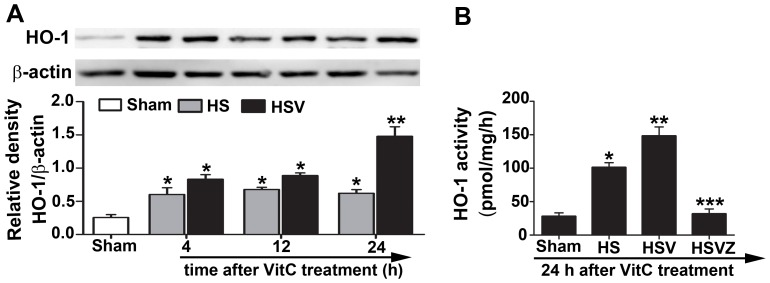
Vitamin C (VitC) enhanced intestinal heme oxygenase (HO)-1 protein level and activity after hemorrhagic shock (HS). Sham: the rats treated with normal saline (NS) and the sham operation; HS: the rats treated with NS and the HS operation; HSV: the rats treated with VitC and the sham operation; HSVZ: some rats in the HSV group additionally treated with Znpp-IX. A, western blot analysis for the time-course change of the intestinal HO-1 protein level in the HS rat treated with NS or VitC. Densitometric analysis was used to calculate the normalized protein ratio (HO-1 to β-actin). B, The HO-1 activity was measured at 24 hours after VitC pretreatment. Data are mean ± SEM, n = 6/group, *p<0.05 compared to sham, **p<0.05 compared to HS at the identical time point, ^***^ *p*<0.05 compared to the HSV.

We investigated the effect of VitC on the HO-1 expression and activity in an HS model of SD rats. Significant increases of the intestinal HO-1 protein were observed in the HS treated rats. In particular, compared to NS, VitC pretreatment led to a significantly higher expression of HO-1 protein in the HS treated rats at 24 hours after pretreatment (Fig.**5A**), and the HO-1 activity exhibited the same trend as the HO-1 protein level at the same time point (**Fig. 5B**). The increased HO-1 activity was diminished to the baseline level after the addition of Znpp-IX.

### VitC pretreatment relieved HS related histological injuries of the intestine via the induction of HO-1

**Figure 6:**
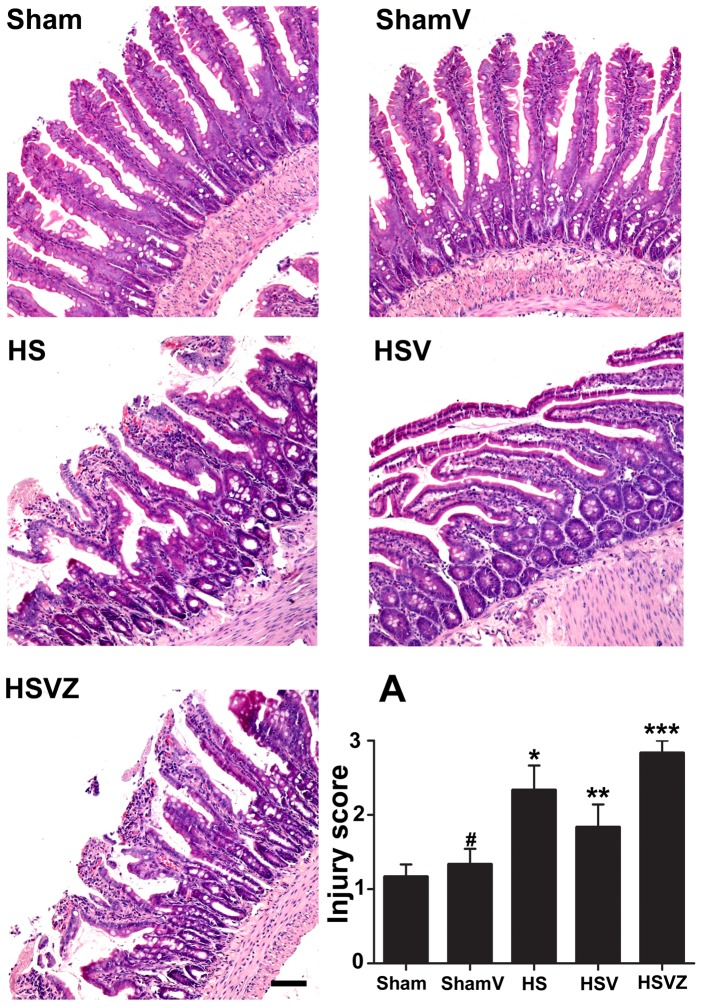
Vitamin C (VitC) pretreatment relieved hemorrhagic shock (HS) related histological intestinal injuries via the induction of heme oxygenase (HO)-1. Sham: the rats treated with normal saline (NS) and the sham operation; ShamV: the rats treated with VitC and the sham operation; HS: the rats treated with NS and the HS operation; HSV: the rats treated with VitC and the sham operation; HSVZ: some rats in the HSV group additionally treated with Znpp-IX. The intestinal samples obtained at 24 hours after VitC pretreatment were stained with hematoxylin and eosin. Magnification: 100X. Scale bar: 200 µm. A, the injury score of all rats. Data are mean ± SEM, n = 6/group, *p<0.05 compared to Sham, **p<0.05 compared to HS, ***p<0.05 compared to HSV.

We then investigated whether HO-1 induction by VitC was responsible for its amelioration of intestinal injuries in HS. Our results showed that obvious pathological damages, including epithelial edema, villi necrosis, and hemorrhaging were present in HS group. Compared to HS group, the tissue damages in HSV group were significantly relieved at 24 hours after VitC pretreatment, which was pronounced again after the HO-1 activity was inhibited by Znpp-IX in HSVZ group. No obvious tissue damage was shown in the rats of ShamV group. The histological changes are demonstrated accurately by the injury score (**Fig. 6A**).

### VitC pretreatment decreased inflammatory response in the intestine via the induction of HO-1

**Figure 7:**
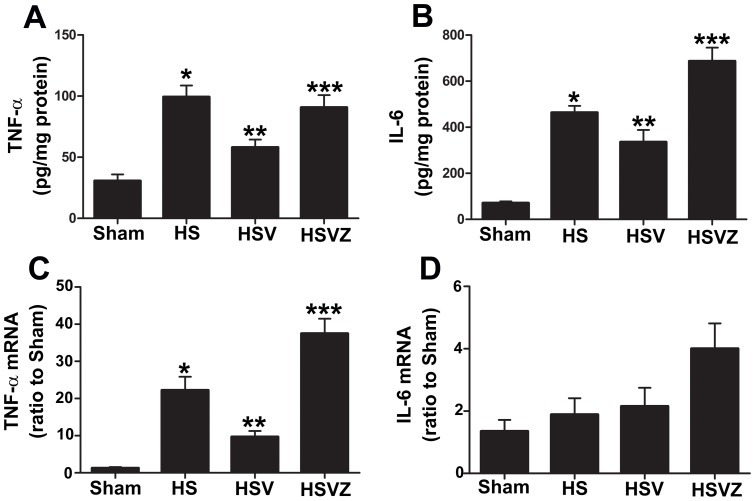
Vitamin C (VitC) pretreatment decreased hemorrhagic shock (HS) related intestinal inflammatory response via the induction of heme oxygenase (HO)-1. Sham: the rats treated with normal saline (NS) and the sham operation; HS: the rats treated with NS and the HS operation; HSV: the rats treated with VitC and the sham operation; HSVZ: some rats in the HSV group additionally treated with Znpp-IX. The intestinal samples were obtained at 24 hours after VitC pretreatment. A and B, the protein levels of the tumor necrosis factor (TNF)-α and interleukin (IL)-6 were evaluated by the enzyme linked immunosorbent assay (ELISA). C and D, the mRNA level of TNF-α and IL-6 were evaluated by real-time PCR. Data are mean ± SEM, n = 6/group, *p<0.05 compared to Sham, **p<0.05 compared to HS,***p<0.05 compared to HSV.

Then we explored how the HO-1 induction by VitC prevented HS related intestinal injuries. Our results showed that the intestinal level of TNF-α protein (**Fig. 7A**) and mRNA (**Fig. 7C**), as well as IL-6 protein (**Fig. 7B**) were significantly elevated in HS group at 24 hours after NS pretreatment compared to Sham group. These increased levels of pro-inflammatory cytokines were down-regulated in HSV group and became pronounced again after the blockage of HO-1 activity by Znpp-IX in HSVZ group. The level of the IL-6 mRNA in all groups demonstrated no significant difference (**Fig. 7D**).

### VitC pretreatment inhibited neutrophil infiltration in the intestine via the induction of HO-1

**Figure 8:**
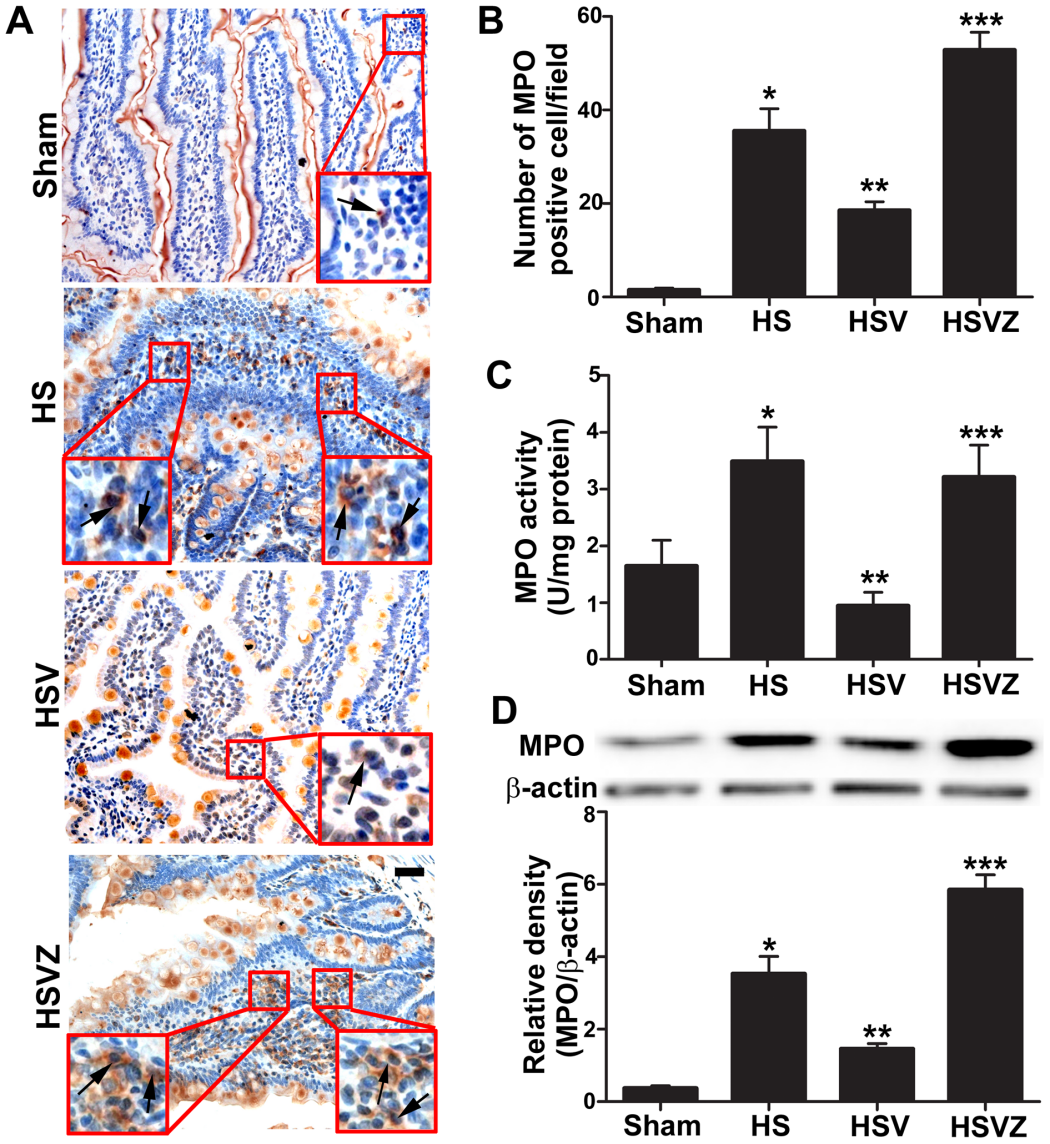
Vitamin C (VitC) pretreatment reduced hemorrhagic shock (HS) related neutrophil infiltration in the intestine via the induction of heme oxygenase (HO)-1. Sham: the rats treated with normal saline (NS) and the sham operation; HS: the rats treated with NS and the HS operation; HSV: the rats treated with VitC and the sham operation; HSVZ: some rats in the HSV group additionally treated with Znpp-IX. The intestinal samples were obtained at 24 h after VitC pretreatment. A, immunohistochemical staining for myeloperoxidase (MPO). The arrows indicate the MPO positive cells. Magnification: 200X. Scale bar: 100 µm. B, the number of the MPO positive cells. C, MPO activity. D, western blot analysis of the MPO protein level. The densitometric analysis was used to calculate the normalized protein ratio (MPO to β-actin). Data are mean ± SEM, n = 6/group, *p<0.05 compared to Sham, **p<0.05 compared to HS, ***p<0.05 compared to HSV.

Pro-inflammatory cytokines such as TNF-α and IL-6 play an important role in neutrophil activation and infiltration as they are required for the adhesion molecule expression and chemokine (such as cytokine-induced neutrophil chemoattractant [CINC] 1 and CINC-3) production [29], [30]. Therefore, we tested the effects of VitC on neutrophil infiltration. Most of the MPO (a marker of neutrophil content) positive cells were observed in the intestinal lamina propria (**Fig. 8A**), and the indicators for neutrophil infiltration, including the number of MPO positive cells (**Fig. 8B**), the MPO activity (**Fig. 8C**) and the protein level (**Fig. 8D**) were significantly pronounced in HS group at 24 hours after NS pretreatment and were observed to be down-regulated in HSV group. The inhibiting effect of VitC on neutrophil infiltration was attenuated after the addition of Znpp-IX in HSVZ group.

### VitC pretreatment reduced HS related apoptosis in the intestine via the induction of HO-1

**Figure 9:**
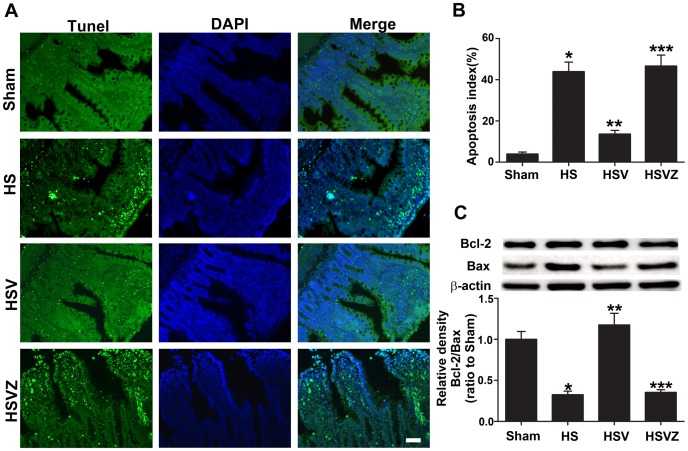
Vitamin C (VitC) pretreatment reduced hemorrhagic shock (HS) related apoptosis in the intestine via the induction of heme oxygenase (HO)-1. Sham: the rats treated with normal saline (NS) and the sham operation; HS: the rats treated with NS and the HS operation; HSV: the rats treated with VitC and the sham operation; HSVZ: some rats in the HSV group additionally treated with Znpp-IX. The intestinal samples were obtained at 24 h after VitC pretreatment. A, the apoptotic cells stained by the TdT-mediated dUTP nick end labeling (TUNEL, green) technique. Magnification: 200X. Scale bar: 100 µm. B, the percentage of the TUNEL positive cells of all the groups expressed as the apoptosis index. C, western blot analysis of the Bcl-2 and Bax protein levels. The densitometric analysis was used to normalize Bcl-2/Bax level by β-actin. The Bcl-2/Bax level was demonstrated as a ratio compared to Sham. Data are mean ± SEM, n = 6/group, *p<0.05 compared to Sham, **p<0.05 compared to HS, ***p<0.05 compared to HSV.

Containing the NADPH oxidase, the infiltrated neutrophil is the major source of ROS and directly causes apoptosis [31]. Therefore, we tested the effect of VitC on HS related apoptosis. Our results showed a significant increase in the number of TUNEL positive cells (**Fig. 9A**) and the apoptosis index (**Fig. 9B**) in HS group compared to Sham group, which was reduced at 24 hours after VitC pretreatment in HSV group. The apoptotic effects were tightly regulated by the balance between the anti-apoptotic protein Bcl-2 and the pro-apoptotic protein Bax, and the ratio between Bcl-2 and Bax is a pivotal factor determining the apoptosis burden [32]. Western blot analysis (**Fig. 9C**) showed that the Bcl-2/Bax ratio was significantly reduced in HS group compared to Sham group, and was up-regulated at 24 hours after VitC pretreatment in HSV group. All the anti-apoptotic effects of VitC were attenuated after the addition of Znpp-IX in HSVZ group.

## Discussion

This study showed that VitC induced HO-1 expression in intestinal epithelial cells via the activation of the ERK1/2 signaling pathway. Pretreatment with VitC significantly alleviated HS related pathological intestinal damage, and this organ protective effect might be related to the attenuation of the inflammatory response, the neutrophil infiltration and the apoptosis. The beneficial effects of VitC pretreatment were abolished after the administration of Znpp-IX, a specific competitive inhibitor of HO-1 activity. These findings provide further evidences of the mechanism by which VitC exert organ protective effects towards I/R injuries.

Our results showed that VitC induced intestinal HO-1 expression both in vitro (**Fig. 3B, C, D** **; Fig. S1**) and in vivo (**Fig. 4A, C**). These results are consistent with other studies which demonstrated the inducing property of VitC on HO-1[14], [15]. HO-1 is usually induced by stress factors such as ROS, heavy metal exposure, and hypoxia [33]. At high dose, VitC has been shown to act as a pro-oxidant that generates ROS [11], [34]. Therefore, the adverse pro-oxidant effects of VitC may be of some concern. However, Chen et al showed [34] that the dose of VitC required for adverse pro-oxidant effects is several times those used in our study (100 mg/kg of body weight) and generated cytotoxicity only in cancer cells, not normal cells. Furthermore, the dose of human equivalent to 100 mg/kg of rat used in this study is approximately 16.2 mg/kg [35], and Tanaka, et al [36] reported the severe burn patients treated with high dose of VitC (66 mg/kg/hour) which was several folds of ours showed no obvious adverse events. Our data of CCK-8 assay (**Fig. 3A**) and pathological evaluation (**Fig. 6**) showed the dose of VitC in vitro (100 µM) and in vivo (100 mg/kg of body weight) is non-toxic to the IEC-6 cell or the sham rats. Therefore, it is concluded that VitC could induce HO-1 without causing any additional adverse effects, which suggests that VitC pretreatment might realize “organ preconditioning” in a relatively safe manner.

**Figure 10:**
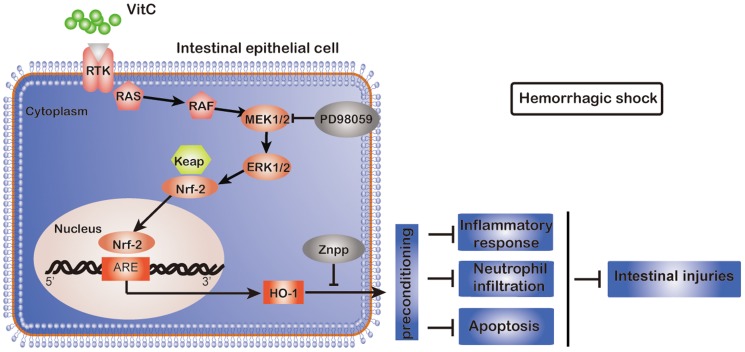
The possible mechanisms by which vitamin C (VitC) pretreatment reduces intestinal injury in hemorrhagic shock (HS). VitC activates the classic ERK1/2 signaling pathway and causes the dissociation of Nrf2 from Keap1, which activates HO-1 gene expression after nuclear translocation by binding to the HO-1 AREs. The over-expression of HO-1, known as “preconditioning”, protects against HS related intestinal injury by inhibiting the inflammatory response, neutrophil infiltration and apoptosis. Legend: RTK, receptor tyrosine kinase; RAS, synaptic Ras-GTPase-activating protein; RAF (known as MAPK3), mitogen-activated protein kinase (MAPK); MEK (known as MAPK2); MAPK kinase; ERK1/2, extracellular signal-regulated kinase; Keap1, Kelch-like ECH-associated protein 1; Nrf2, NF-E2-related factor 2; ARE, antioxidant response element; HO-1, heme oxygenase-1; Znpp-IX, zinc protoporphyrin-IX.

Three major subfamilies of MAPK, including p38MAPK, ERK1/2 and JNK, have been regarded as important pathways for the induction of HO-1 gene expression [9]. Previous studies have shown different MAPK pathways involved in mediating HO-1 induction in different types of cells under different stimuli. For example, the ERK1/2 and p38MAPK pathways were shown to be involved in the induction of HO-1 by arsenite in hepatoma cells [37] and only the p38MAPK was shown to mediate the induction of HO-1 by IL-10 in murine macrophages [38]. We showed that VitC led to a rapid phosphorylation of p38MAPK, ERK1/2 and JNK (**Fig. 3E**), and only the inhibitor of ERK1/2, PD98059, could inhibit the VitC induced HO-1 expression (**Fig. 3F**). Our findings are inconsistent with the recent findings of Huang et al. [14], which showed that p38MAPK rather than ERK1/2 mediated the induction of HO-1 via VitC in glial and neuronal cells. Therefore, different MAPK subfamilies may be responsible for mediating the HO-1 induction by VitC in different cell types. In addition, ERK1/2 has been shown to be an up-streaming regulator for the nuclear translocation of the NF-E2-related factor (Nrf)-2, a key transcription factor regulating HO-1 expression [39]. To be consistent with this notion, it might be speculated that VitC activates ERK1/2, and in turn, promotes Nrf-2 mediated HO-1 expression (**Fig. 10**). Furthermore, the activation of ERK1/2 has been recently shown to mediate several other roles of VitC beside the HO-1 induction, such as promoting the proliferation of adipose-derived stem cells [40] and inducing periodontal ligament progenitor cell differentiation [41].

In this study, we used a fixed pressure model of HS and the MAP level is regarded as the main indicator. During the establishment of HS model, the MAP value was decreased to 30.3±5.6 mmHg (**Fig. 2A**) which had been maintained for 1 hour. This MAP value actually met the requirement for the HS model establishment. This pattern of fixed pressure was applied in other studies of HO-1 expression in organs of HS model [20], [21]. For example, Inoue et al [20] recently showed that HO-1 was induced in a site-specific pattern of high expression in the duodenum, jejunum, and colon, but is barely detected in the ileum in HS models of rat. These results were consistent with our findings as we showed that HO-1 protein level (**Fig. 5A**) and activity (**Fig. 5B**) in the jejunum were significantly induced after HS.

Inoue et al. [20] also showed the intestinal section where HO-1 expressed more suffered less tissue injuries after HS and concluded that HO-1 exerted a protective effect. Consistent with their findings, we showed that VitC pretreatment led to a further enhancement of HO-1 protein level and activity **(** **Fig. 5** **)** after HS, and that the intestinal injuries in HS rats diminished concurrently. The improvement of intestinal injuries was abrogated after the inhibition of HO-1 by Znpp-IX. These findings suggested that VitC pretreatment might further enhance the HO-1 expression in the jejunum to an extent which are adequate to reduce the HS related injuries. These “further enhancing” effects were also demonstrated by other agents including hemin [8] and estrogen [29], which were shown to be protective against oxidative injuries. Therefore, it is speculated that VitC pretreatment improved the HS related injuries in the jejunum not only due to the pre-induction of HO-1 realizing “organ preconditioning”, but also by maintaining longer and higher HO-1 over-expression than the endogenous HO-1 induction by HS per se. This notion may partly elucidate the recent findings [13] that resuscitation with VitC alleviated HS related injuries and inflammatory responses.

As an innate immune effector, the intestinal epithelial cell has been shown to secrete pro-inflammatory cytokines in the early stage of HS [42]. Pro-inflammatory cytokines (such as TNF-α and IL-6) are required for the adhesion molecule expression and chemokine (such as cytokine-induced neutrophil chemoattractant [CINC]-1 and CINC-3) production [29], [30], which are essential for neutrophil activation and infiltration. Infiltrated neutrophils are the major source of ROS, which directly cause apoptosis [39]. Furthermore, ROS was shown to serve as a second messenger for pro-inflammatory signaling cascades [43]. As a result, a vicious cycle might exist in the initiating stage of HS that eventually leads to tissue injuries, and the inflammatory response derived from mucosal epithelial cells might be the initiator. Our immunohistochemistry results showed that HO-1 signals were predominantly observed in the mucosal epithelial cells (**Fig. 4C**). Furthermore, the immunofluorescence results showed that the VitC-induced HO-1 expression was mainly located in the cytoplasm of IEC-6 cell (**Fig. 3D**) which indicated the functional compartmentalization of HO-1 [44]. Therefore, it is speculated that the organ protective mechanism of VitC pretreatment might be associated with the inhibition of the initial inflammatory response in the epithelial cells via the induction of anti-inflammatory HO-1 [9].

In conclusion, we showed that pretreatment with VitC led to the attenuation of intestinal injuries in HS, and the underlying mechanism for this was probably related to the pre-induction of HO-1 without causing any adverse effects and the longer and higher maintaining of HO-1 expression after HS. These findings may provide an easy and safe method for clinical “organ preconditioning”, which may be applicable in the clinical situations where hemorrhage is foreseeable, such as prior to major surgery.

## Supporting Information

Figure S1 **Vitamin C (VitC) induced Heme oxygenase (HO)-1 expression in IEC-6 intestinal epithelial cell.** The IEC-6 cells were treated with treated NS (Control) or VitC for 24 hours (hrs), the immunocytochemsity was performed using DAB staining. The brown staining indicated HO-1 expression.
(PDF)

Table S1 **Sequences of the upstream and downstream primers used in this study.**
(PDF)

Checklist S1 **The ARRIVE Guidelines Checklist.**
(PDF)
