# Supplementary material for: Pharmacological Preconditioning with Vitamin C Attenuates Intestinal Injury via the Induction of Heme Oxygenase-1 after Hemorrhagic Shock in Rats
Source: PLoS One. 2014 Jun 13;9(6):e99134. doi: 10.1371/journal.pone.0099134 (PMC4057195; doi:10.1371/journal.pone.0099134)
Supplement: Figure S1 — Vitamin C (VitC) induced Heme oxygenase (HO)-1 expression in IEC-6 intestinal epithelial cell. The IEC-6 cells were treated with treated NS (Control) or VitC for 24 hours (hrs), the immunocytochemsity was performed using DAB staining. The brown staining indicated HO-1 expression. (PDF) [file pone.0099134.s001.pdf]

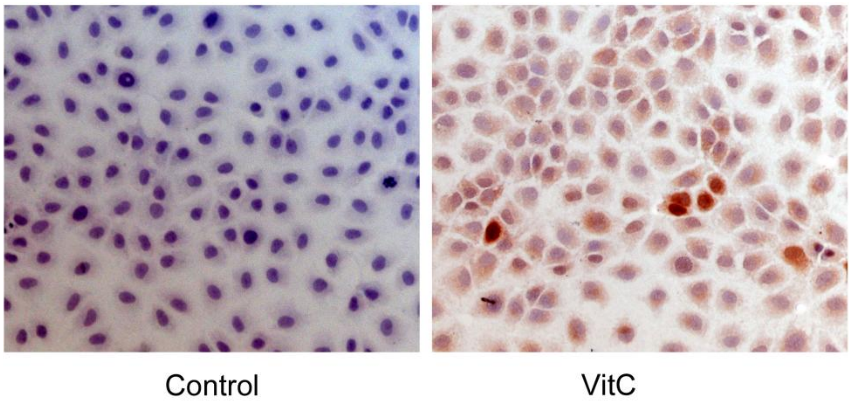

Supplement 2. Vitamin C (VitC) induced heme oxygenase (HO)-1 expression in IEC-6 intestinal epithelial cell. The IEC-6 cells were treated with treated NS (Control) or VitC for 24 hours (hrs), the immunocytochemsity was performed using DAB staining. The brown staining indicated HO-1 expression.
