# Supplementary material for: Pharmacological Preconditioning with Vitamin C Attenuates Intestinal Injury via the Induction of Heme Oxygenase-1 after Hemorrhagic Shock in Rats
Source: PLoS One. 2014 Jun 13;9(6):e99134. doi: 10.1371/journal.pone.0099134 (PMC4057195; doi:10.1371/journal.pone.0099134)
Supplement: Table S1 — Sequences of the upstream and downstream primers used in this study. (PDF) [file pone.0099134.s002.pdf]

Table 1. Sequences of the upstream and downstream primers used in this study.

Gene	Primer Sequences (5'→3')	
TNF-α	upper	CCCAATCTGTGTCCTTCTAACT
	lower	CACTACTTCAGCGTCTCGTGT
IL-6	upper	CAAAGCCAGAGTCATTCAAGC
	lower	GGTCCTTAGCCACTCCTTCTGT
β-actin	upper	GCGCTCGTCGTCGACAACGG
	lower	GTGTGGTGCCAAATCTTCTCC
